# Multimodel Integrated Enterprise Credit Evaluation Method Based on Attention Mechanism

**DOI:** 10.1155/2022/8612759

**Published:** 2022-02-21

**Authors:** Lei Zhang, Qiankun Song

**Affiliations:** ^1^School of Economics and Management, Chongqing Jiaotong University, Chongqing 400074, China; ^2^School of Mathematics and Statistics, Chongqing Jiaotong University, Chongqing 400074, China

## Abstract

Due to the difficulty of credit risk assessment, the current financing and loan difficulties of small- and medium-sized enterprises (SMEs) are particularly prominent, which hinders the operation and development of enterprises. Based on the previous researches, this paper first screens out features by correlation coefficient method and gradient boosting decision tree (GBDT). Then, with the help of SE-Block, the attention mechanism is added to the feature tensor of the subset separated from metadata. On this foundation, two models, XGBoost and LightGBM, are used to train four subsets, respectively, and Bayesian ridge regression is used to fuse the training results of single models under different subsets. In the simulation experiment, the AUC value of the NN-ATT-Bayesian-Stacking model reaches 0.9675 and the distribution of prediction results is ideal. The model shows good robustness, which could make a reliable assessment for the financing and loans of SMEs.

## 1. Introduction

According to statistics, by the end of 2018, the number of SMEs in China has exceeded 30 million, accounting for more than 50% of the national tax [1], playing an increasingly important role in national economic development and scientific innovation. However, the financing and loan difficulties of SMEs are particularly prominent, and they are facing severe and complex risks and challenges. In this context, the establishment of a scientific and effective credit and risk assessment method is of great significance to the development of SMEs and the industry regulation of banks and financial services.

Credit evaluation models and subset selection models can be divided into traditional statistical models and machine learning models [2–5]. The earliest credit evaluation models are generally based on classical statistical theory, including methods such as logistic regression, linear regression, and naive Bayes classification. In recent years, with the improvement of computing power, the rapid progress of algorithms, and the substantial increase of data size, methods in fields like machine learning and deep learning become more and more popular in enterprise credit evaluation [6–8]. The representative data mining models include support vector machine (SVM), K-Means, decision tree, artificial neural network, and long-term and short-term memory artificial neural network [9, 10]. At the same time, ensemble-learning methods have also achieved good results in credit evaluation problems. Huang et al. [11] compare the effects of several commonly used neural network models for credit evaluation in the SME data set. The results show that the Probabilistic Neural Network (PNN) has the smallest error rate and the second type of error and possesses the highest AUC value and strong robustness. Cai et al. [12] construct a supply chain risk evaluation index system and a supply chain risk evaluation model based on BP neural network, which has unique advantages in solving highly nonlinear problems such as supply chain risk evaluation and provides a good reference for enterprises and other financial institutions to expand supply chain financing business. Wang et al. [13] propose a credit assessment method of the long-short-term memory network and attention mechanism (ATT-LSTM). Event2vec model is constructed to convert each type of event into a vector, and then the LSTM network with attention mechanism is used to predict the default probability of users.

Compared with the traditional manual feature extraction method and the standard LSTM model, this method can effectively improve recognition accuracy. Zhang et al. [14] construct a credit risk assessment method based on deterministic annealing semisupervised support vector machine (DAS3VM), which obtains good results in sparse and asymmetric data sets. Luo et al. [15] propose a new unsupervised kernelless quadric support vector machine (QSSVM) model, which avoids the selection of kernel and related kernel parameters, and the golden section algorithm is designed to generate appropriate classifiers for balanced and unbalanced data.

Based on single models, attribute partition and multimodel fusion are the focuses of current research. Addo P *M* et al. [16] construct a binary classifier based on machine and deep learning models or real data to predict the probability of loan default. The most important 10 features are further selected from these models, and the stability of the binary classifier is tested by comparing the performance of the binary classifier. Zhu et al. [17] combine two classical integrated ML methods: random subspace (RS) and Multiboosting and propose an enhanced hybrid integrated ML method RS-Multiboosting to improve the accuracy of credit risk prediction of SMEs. Diego [18] constructs LR model, ANN, and three two-stage hybrid models integrated with LR and ANN on the basis of logistic regression and ANN, and the effectiveness of the two-stage hybrid model is verified by simulation experiments. Tian [19] proposes a gradient boosting decision tree. The algorithm adopts a new data preprocessing and feature selection method, which performs well in classification generalization. Arora N [20], based on the Bolasso algorithm, establishes a BS-RF credit risk assessment model. Bolasso algorithm provides significant feature stability for RF, and BS-RF model shows good recognition accuracy, which is superior to other methods in AUC and accuracy. Liu [21] puts forward a new multiobjective soft subspace clustering algorithm for credit risk assessment. Firstly, a soft subspace clustering validity index for credit risk assessment is established, which can detect the underlying subspace of each clustering from the whole high dimensional feature space and thus obtain a complete data structure in the clustering process. Secondly, a multiobjective evolutionary algorithm is proposed to optimize the clustering criterion of the new clustering validity index. There is no predefined weighting coefficient, which ensures the robustness of the algorithm. Lappas [22] uses a strategy of combining soft computing method with expert knowledge. Through the participation of experts in the credit scoring process, the ability of explaining the prediction ability of each feature in the credit data set is strengthened, and a feature selection method based on wrapper is then proposed. Hou [4] puts forward an improved synthetic minority oversampling technique method. On this basis, training and learning are carried out by combining the long-short-term memory network and the adaptive AdaBoost algorithm, which can achieve good results in the problem of unbalanced credit risk evaluation.

Compared with the single model evaluation method, the multimodel integrated method performs better, but the above models and methods in attribute selection mostly simply eliminate the redundant features in metadata and put a subset into one or more base models for training, instead of comparing the results of selected subset based on different base models. On the foundation of the above research, this paper combines different subset partition methods (the correlation coefficient and GBDT) to divide the subset of the original data set. Then, the random forest model is used to carry out the model verification of the divided subset, which ensures the validity and integrity of partitioned data. To further enhance the model's attention to important features, this paper adds a channel attention mechanism to feature tensor and further applies XGBoost and LightGBM to train four subsets with attention mechanism module, respectively, based on SE-Block. Finally, Bayesian ridge regression is used to fuse the training results of single models under different subsets and output the final evaluation results. In the simulation experiment, the AUC value of the NN-ATT-Bayesian-Stacking model established in this paper reaches 0.9675, and the distribution of prediction results is ideal, which shows good robustness and can make reliable assessments for financing and loan of SMEs. The main contributions of this paper are as follows:

1. A multimodel fusion enterprise credit evaluation framework based on attention mechanism is constructed, which has important practical significance and theoretical value.
2. On the basis of SE-Block, the channel attention mechanism is added to the feature tensor obtained by feature engineering, which improves the attention of the model. The effectiveness of the attention mechanism is verified in the simulation experiment.
3. This paper first uses the correlation coefficient and GBDT to divide the original data set into subsets and uses the random forest to verify the effectiveness of the divided subsets. On this basis, two ensemble-learning models: XGBoost and LightGBM are used to train the four subsets, respectively. Finally, Bayesian ridge regression is used for model fusion, which significantly improves the effect of credit evaluation.
4. In the simulation experiment, one or more subsets are put into one or several base models for training, and the results of selecting subsets are compared and analyzed based on different base models.

## 2. Base Model Module

### 2.1. Random Forest

The operation of Bagging integration is that, for a given data set containing *m* samples, a sample is randomly taken out and put into the sampling set, then the sample is put back to the initial data set, and then the operation is repeated several times. After *m* cycles, the sample set of *m* samples is obtained. In the process of multiple sampling, a sample has a chance to be selected multiple times.

Random forest is an extension of Bagging. Based on Bagging ensemble with decision tree as the base learner, RF further introduces random attribute selection in the training process of decision tree. Specifically, the traditional decision tree selects an optimal attribute at the current node when selecting attributes for division. However, while RF divides each node of the base decision tree, it first randomly selects an attribute subset that contains *k* attributes from the current node and then selects an optimal attribute from this subset to construct a partition node.

The random forest model is a variance-based model, so the prediction results are difficult to fit. However, it can be used to test the validity of feature subset division. On this basis, this paper applies random forests to test whether the partition error of subsets is large.

### 2.2. Decision Tree Model

Decision tree and its advanced model are algorithms that divide input space into different regions and each region has independent weight parameters. In machine learning, decision tree is a prediction model, which represents a mapping relationship between object attributes and object values. Each node in the tree represents a feature; that is, any node represents a small solution space. Each branch path represents a possible attribute value, that is, node weight. And each leaf node denotes the attribute value of the object represented by the path from the root node to the leaf node.

Let ${\hat{y}}_{i}^{\left(t\right)}$ be the prediction value of the *i* th example in the *t* th tree, let *ω*(*f*_*t*_) be the size of the *t* th tree, let *f*_*t*_(*x*_*i*_) be the weight value of the leaf node corresponding to the *i* th training sample of the *t* th tree, and let *L*^(*t*)^ be the objective function for tree *t* as shown in

(1) $$\begin{array}{c} L^{\left(t\right)}=\sum_{i=1}^{n}l\left(y_{i},{\hat{y}}^{\left(t-1\right)}+f_{t}\left(x_{i}\right)\right)+\omega \left(f_{t}\right), \end{array}$$

Using Taylor Quadratic Expansion for *f*_*t*_(*x*_*i*_), further we can get

(2) $$\begin{array}{c} L^{\left(t\right)}≃\sum_{i=1}^{n}\left[l\left(y_{i},{\hat{y}}^{\left(t-1\right)}\right)+g_{i}f_{t}\left(x_{i}\right)+\frac{1}{2}h_{i}f_{t}^{2}\left(x_{i}\right)\right]+\omega \left(f_{t}\right). \end{array}$$

Among them, *g*_*i*_ is the first-order gradient of the loss function to ${\hat{y}}_{i}^{\left(t-1\right)}$, and *h*_*i*_ is the second-order gradient of the loss function to ${\hat{y}}_{i}^{\left(t-1\right)}$. Derived from the formula, the loss function value of the *t* − 1 trees before the *t* th tree is known, and ${\tilde{L}}^{\left(t\right)}$ represents the approximate value; so,

(3) $$\begin{array}{c} {\tilde{L}}^{\left(t\right)}=\sum_{i=1}^{n}\left[g_{i}f_{t}\left(x_{i}\right)+\frac{1}{2}h_{i}f_{t}^{2}\left(x_{i}\right)\right]+\omega \left(f_{t}\right). \end{array}$$

Define *I*_*j*_={*i|x*_*i*_=*j*} as a mapping from the *i* th sample to the *j* th child node; then,

(4) $$\begin{array}{c} {\tilde{L}}^{\left(t\right)}=\sum_{i=1}^{n}\left[g_{i}f_{t}\left(x_{i}\right)+\frac{1}{2}h_{i}f_{t}^{2}\left(x_{i}\right)\right]+\gamma T+\frac{1}{2}\lambda \sum_{j=1}^{T}w_{j}^{2}\\ =\sum_{j=1}^{T}\left[\left(\sum_{i\in I_{j}}g_{i}\right)w_{i}+\frac{1}{2}\left(\sum_{i\in I_{j}}h_{i}+\lambda \right)w_{j}^{2}\right]+\gamma T. \end{array}$$

Further, the first derivative of *w*_*j*_ for ${\tilde{L}}^{\left(t\right)}$ is set to 0, so we can obtain

(5) $$\begin{array}{c} w_{j}^{*}=-\frac{\sum_{i\in I_{j}}g_{i}}{\sum_{i\in I_{j}}h_{i}}. \end{array}$$

Then, bring *w*_*j*_^*∗*^ back to the above formula:

(6) $$\begin{array}{c} {\tilde{L}}^{\left(t\right)}=-\frac{1}{2}\sum_{j=1}^{T}\frac{{\left(\sum_{i\in I_{j}}g_{i}\right)}^{2}}{\sum_{i\in I_{j}}h_{i}+\lambda }+\gamma T. \end{array}$$

Therefore, the solution of the objective function can be obtained.

### 2.3. Module of Channel Attention Mechanism Based on SE-Block

The original intention of Squeeze-and-Excitation Networks (SE-Block) is to solve the problem of feature loss caused by the different importance of each channel in the Conv-Pool process of CNN. In the traditional CNN feature extraction network, feature extraction mainly relies on convolution operation to combine spatial information and channel information in the local receptive field, and by default each channel of feature map generated at each layer is equally important. However, in practical problems, the importance of channels may be significantly different, and even there may be a certain dependence between different channels.

**Figure 1:**
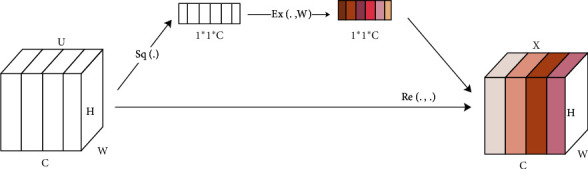
Network construction of SE-Block module.

The structure of *SE-Block* module is shown in Figure 1. For the feature map with *C* feature channels, the work of *SE-Block* is divided into three parts:

(1) The first part is the *Squeeze* module. In order to obtain the global receptive field with different feature channels, the module first compresses the input feature map in the channel dimension, and the compression methods include global average pooling and global maximum pooling. The pooling formula is shown in the following formulas. After compression, the two-dimensional feature channels in the feature map are transformed into a real number possessing a channel global receptive field:
  (7) $$\begin{array}{c} z_{c}=F_{sq-\text{avg}}\left({\text{Map}}_{c}\right)\\ =\frac{1}{H\times W}\sum_{i=1}^{H}\sum_{j=1}^{W}{\text{Map}}_{c}\left(i,j\right), \end{array}$$
  (8) $$\begin{array}{c} z_{c}=F_{sq-\max}\left({\text{Map}}_{c}\right)\\ =\underset{\text{0}\leq i<H\text{,0}\leq j<W}{\text{Max}}{\text{Map}}_{c}\left(i,j\right). \end{array}$$
Map is the input feature graph, *H* and *W* are the width and height of the feature graph, respectively.
(2) The second module is the *Excitation* module, which adopts a two-layer fully connected neural network and adds more nonlinear processes to fit the complex correlation between channels. The first layer is a dimension reduction layer with *C*/*r* neurons and ReLU activation function. The number of neurons in the second layer is *C*. The Sigmoid is used to generate 0–1 real numbers, and the output dimension matches the number of channel dimensions. The mapping function of the module is shown in
  (9) $$\begin{array}{c} F_{ex}\left(z,W\right)=\sigma \left(g\left(z,W\right)\right)\\ =\sigma \left(W_{2}\delta \left(W_{1}z\right)\right). \end{array}$$
Among them, *W*_1_ ∈ *ℝ*^(*c*/*r*)×*c*^, *W*_2_ ∈ *ℝ*^*c*×(*c*/*r*)^, *σ* represents ReLU activation function, *δ* represents activation function Sigmoid, and *g* represents nonlinear mapping function.
(3) The third module is *Reweight* module. This module adds importance weight to each channel dimension according to the output of Excitation.

## 3. Feature Selection-Test Feasibility by Random Forest

Filter is a method of feature selection, which scores each feature according to divergence or correlation, sets threshold or the number of thresholds to be selected, and then selects features. In this paper, the steps to use filter are as follows:

**Step 1 .**

The correlation coefficient method: calculate the correlation sparsity between features and select the features possessing a larger Pearson's coefficient according to the threshold.

**Step 2 .**

Directly delete features whose variance does not meet the threshold or delete directly one of two features with larger Pearson's coefficients.

### 3.1. Correlation Coefficient Method

For the correlation between the two attribute features A and B, we can calculate the correlation coefficients of features (also known as Pearson's product moment coefficient) of A and B to obtain the correlation degrees *r*_*A*,*B*_ of these two features:

(10) $$\begin{array}{c} r_{A,B}=\frac{\sum_{i=1}^{n}\left(a_{i}-\bar{A}\right)\left(b_{i}-\bar{B}\right)}{n\sigma_{A}\sigma_{B}}\\ =\frac{\sum_{i=1}^{n}\left(a_{i}-b_{i}\right)nA\bar{B}}{n\sigma_{A}\sigma_{B}}. \end{array}$$

Among them, *n* is the number of tuples, *a*_*i*_ and *b*_*i*_ are the values of tuple *i* on *A*, *B*, respectively, $\bar{A}\bar{B}$ denote the mean of *A* and *B*, *σ*_*A*_, *σ*_*B*_ denote the standard deviation of *A*, *B*, and ∑(*a*_*i*_*b*_*i*_) is the cross product sum of *A*, *B* (for each tuple, use the product within the elements). If *Pearson* is greater than zero, it indicates that one value increases with the increase of another value, that is, a positive correlation. If it is negative, it means that one value decreases with the increase of another. The larger the *Pearson* is, the stronger the correlation is, and the more obvious the data redundancy is.

In order to prevent the features of high correlation, which will cause data redundancy, for the data with a high correlation coefficient, the features of the training data set are divided. The original *N-*dimensional data are divided into *m* subsets, and the union of all subsets is the *N-*dimensional data. Then, the model is trained in turn, and finally the model fusion is carried out to obtain the best data. Split the features with high correlation coefficients into two subsets, and here the metadata is divided into subsets 1 and 2.

The results of random forest test are in Table 1.

Through data comparison, although the accuracy of the subsample set data has a certain decline, the decline is not large and the data redundancy is avoided. Thus, the division can be considered reasonable.

### 3.2. Tree Model Selection Method

Tree model selection is a model-based selection method, rather than a feature selection method based on the basic properties of the numerical value. The tree model is based on the Gini index to divide nodes and get the index value of features.

Assuming that the proportion of class *k* samples in the current sample set *D* is *p*_*k*_(*k*=1,2,…, |*y*|), then the Gini index of *D* is defined as

(11) $$\begin{array}{c} \text{Gini}\left(D\right)=\sum_{k=1}^{\left|y\right|}\sum_{k^{\prime }\neq k}p_{k}p_{k^{\prime }}\\ =1-\sum_{k=1}^{\left|y\right|}p_{k}^{2}. \end{array}$$

Intuitively, Gini(*D*) reflects the probability that two samples are randomly selected from data set *D* with different class labels. Therefore, the smaller the Gini(*D*) is, the higher the data set *D* is raised. So, in the candidate attribute set, select the attribute with the smallest Gini index as the left and right partition attribute.

The metadata is divided into subset 3 and subset 4, where subset 3 is an important feature considered by the GBDT model, and subset 4 is an unimportant feature considered by the GBDT model. Use random forest to test subset.

It can be seen from the data in Table 2 that the accuracy obtained by dividing the two subsets is not much different and it can be considered reasonable. Based on two different methods, the first one is based on mathematical relations, and the second one is based on model selection. Since the results obtained have differences, it is not yet able to determine which method is the best for feature selection. Therefore, a vertical comparison is made for different methods and different subsets to try to achieve higher scores. Meanwhile, a comparison will be carried out with the metadata that is not divided into subsets. And we only use correlation coefficient method and GBDT selection method, which are subset 1, subset 2, subset 3, and subse t4, respectively, in the follow-up. When the sample space is divided, the divided sample space pays more attention to the local sample space of the feature than the global sample space, so the accuracy of the single model will decrease, and the accuracy of the whole model should be ensured not lost. In the following research, the method of *stacking* model fusion is applied to fully use each data and construct a model that can better take care of the global sample.

## 4. Design of Credit Evaluation Model for SMEs

**Figure 2:**
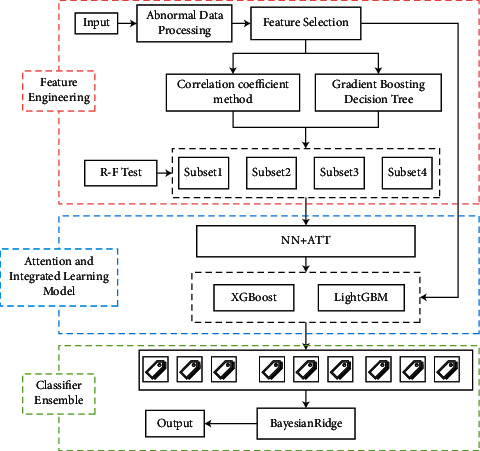
Model structure.

Figure 2 shows the schema diagram for the model used in this paper. After processing the abnormal data and feature engineering, two methods of correlation coefficient and GBDT are used to divide metadata into four channels. On this basis, NN *+* ATT is used to increase the channel attention mechanism to obtain the interaction features in data. As the first prediction of data, different channels correspond to different rough prediction results. Then, the coarse prediction results of corresponding four channels are used as the input features of XGB and LGB to prediction, respectively, and 12 fine prediction results are obtained. Finally, model fusion is performed to obtain the final result.

## 5. Experimental Results and Analysis

### 5.1. Introduction of the Experimental Environment

The model in the experiment is built based on TensorFlow and scikit-learn frameworks, and GPU is used to accelerate computing. The experimental platform and software versions are shown in Table 3.

### 5.2. Data Set Introduction

The data used in this experiment contains the data of 14366 enterprises. Each data item records the features of 274 dimensions including enterprise type, registration authority, enterprise status, total investment, registered capital, industry code, industry category, enterprise category, value-added tax, enterprise income tax, and stamp tax.

### 5.3. Performance Comparison of Different Evaluation Models

In this section, in order to compare the evaluation effects of different models, this paper first uses linear regression, logistic regression, Bayesian ridge regression, and CNN, NN, LGB, and XGB as the basic models for credit risk evaluation. Then, on this basis, NN-ATT, NN- LGB, NN -XGB, CNN-LGB, CNN-XGB, NN-ATT-LGB, NN-ATT-XGB, and NN-ATT-LGB-XGB evaluation models are constructed, compared, and analyzed.

In this paper, each optimization algorithm uses the method of fivefold cross-validation to count the success rate of classification. The data set is randomly divided into five uniform subsets. Each time four subsets are selected to train the model and the remaining one is used to verify the success rate of classification. The mean of five experiments is the success rate of the model under this optimization algorithm.

AUC values for base models, stacked models, and fusion models are shown in Table 4–Table 6.

It can be seen from Table 4 that, in the metadata, the evaluation model with the highest AUC value is the XGB model and the AUC value reaches 0.9285, followed by the LGB model whose AUC value reaches 0.9266. The Bayesian ridge regression model has the lowest AUC value which is 0.8921. Among these four subsets, the models of the top two AUC values are both XGB and LGB, whose AUC values reach 0.92–0.93. The worst is linear regression, whose AUC value is about 0.88. Through the training results of the base model in the metadata set and the subdata set, it can be found that the credit evaluation problem is a relatively complex classification problem and the ordinary linear model is difficult to obtain good results. The two integrated learning methods of XGB and LGB are very effective in such problems.

Table 5 shows the training results of the stacked model constructed in this paper in the metadata set and the subdata set. Compared with the performance of XGB and LGB in Table 4, the improvement of AUC is not very obvious, but it does not mean that NN or CNN is ineffective. The distribution of data changes after adding NN or CNN, which is very important for the later model fusion, will be discussed in detail later.

It can be seen from Table 6 that, in the metadata, the evaluation model with the highest AUC value is the Logistic-Stacking fusion model and the AUC value reaches 0.9673. The second is the Linear-Stacking fusion model, whose AUC value reaches 0.9644, and the AUC value of the fusion model with attention mechanism is about 0.95. In the subdata set, the evaluation model with the highest AUC value is the NN-Logistic-Stacking fusion model, and the AUC value reaches 0.9775. The second is the CNN-Logistic-Stacking fusion model, with AUC value reaching 0.9727. In the fusion model with the addition of attention mechanism, the AUC value of the NN-ATT-Logistic-Stacking fusion model reaches 0.9711, and the AUC values of the other two fusion models based on the attention mechanism are close to 0.97. The fusion models have achieved good results. On the basis of attribute selection and subset division, the performance of the divided training model is significantly better than that of the training model in the metadata set. On this basis, this paper will further discuss and analyze the distribution of the data in the future.

**Figure 3:**
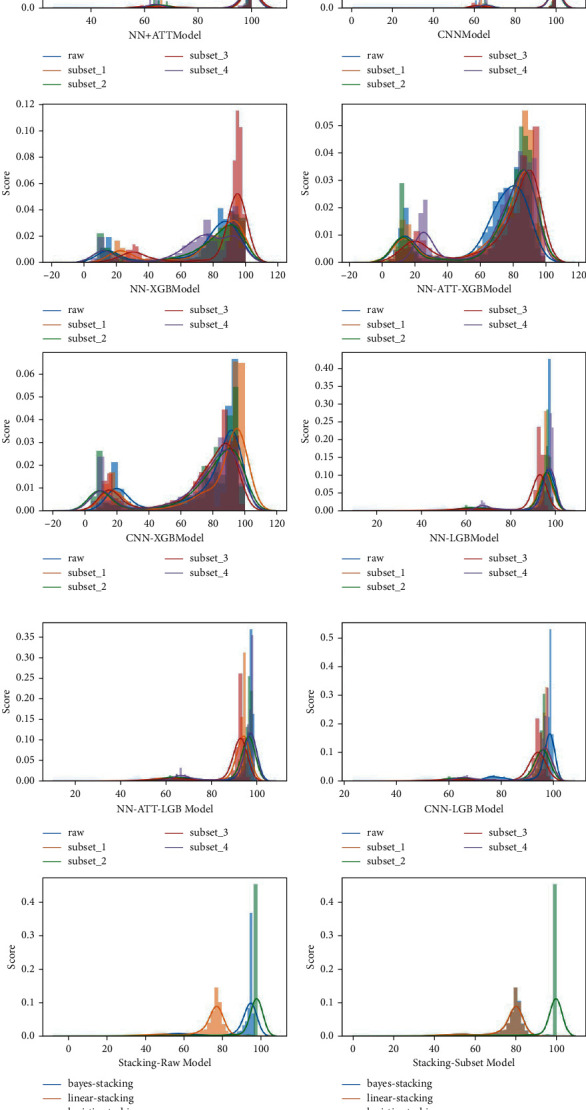
Distribution map of prediction by evaluation model.

The data distribution of the model to the enterprise credit evaluation score is shown in Figure 3. The evaluation model constructed by Bayesian ridge regression is relatively close to the data distribution of metadata and subsets 1 and 3, which is concentrated near 84 points, but the overall coverage and the distribution of prediction results in subsets 2 and 4 are relatively poor. The evaluation model constructed by linear regression has poor performance in metadata and all subdata sets and cannot complete the evaluation task. The four evaluation models constructed by logistic regression, LGB model, NN model, and NN-ATT model have an ideal overall distribution in the metadata and all sub-data sets, but the prediction results of the models are relatively concentrated around 99 points and need to be improved. The overall distribution of the evaluation models constructed by the XGB model is relatively scattered, and the concentrated area of distribution is reasonable, but the amount of data between 20 and 40 points is small, showing a bimodal distribution. The evaluation model constructed by the CNN model has an ideal distribution of prediction results in subset 3, but the distribution of prediction results in metadata and subsets 1, 3, and 4 is too concentrated in 96 points, which needs to be improved.

By analyzing the data distribution of the prediction results of the base model, it can be found that the performance of each model in different data sets highlights the corresponding characteristics of the model and no evaluation model performs well in all aspects.

In the stacking model, compared with the XGB model, the stacking structure of the NN model improves the original bimodal distribution, making the distribution of the prediction results more concentrated around 90 points. In the NN-ATT-XGB and CNN-XGB models, the stacking structure makes the original prediction distribution more concentrated. The NN-ATT-LGB model, the NN-LGB model, and the CNN-LGB model, to a certain extent, solve the problem of overcentralized distribution of the prediction results of the base model, and the distribution is more reasonable. However, the three stacked models have their own advantages and differences in different data sets, which creates conditions for subsequent model fusion.

In the distribution of fusion model prediction results, the distribution of data can be roughly divided into two categories. One type of data is distributed around 100 points; the other type of data is distributed around 80 points. It can be considered that the fusion model near 100 points is a failure. Even if there is a relatively high AUC value, it cannot make a reasonable prediction in the actual credit evaluation.

On this basis, the relatively reasonable models are the Linear-Stacking model trained in metadata set and Linear-Stacking model, Bayesian-Stacking model, CNN-Bayesian-Stacking model, CNN-Linear-Stacking model, NN-Bayesian-Stacking model, NN-Linear-Stacking model, and NN-ATT-Bayesian-Stacking model trained in subset.

Considering the AUC value and the distribution of data, the AUC value of the NN-ATT-Bayesian-Stacking model reaches 0.9675, and the distribution of prediction results seems to be ideal. Thus, this model is the optimal credit evaluation model obtained in this paper.

## 6. Conclusions

In recent years, with the strong support of national policy, small- and medium-sized enterprises have developed vigorously, and their supporting role in the national economy becomes more and more obvious. However, due to the difficulty of credit risk assessment, the financing and loan difficulties of small- and medium-sized enterprises are particularly prominent, which hinder the operation and development of enterprises.

With the improvement of computer computing power, the rapid progress of algorithms, and the increase of data scale, methods in the fields of machine learning and deep learning have shined brightly in the problem of the corporate credit assessment. Based on previous research, this paper constructs a dual model fusion algorithm based on feature selection and channel attention mechanism. The algorithm uses the correlation coefficient method and GBDT to filter the original features and then adds the attention mechanism to the feature tensor of the separation subset of the metadata set based on SE-Block. On this basis, XGBoost and LightGBM are used to train four subsets, respectively. Finally, Bayesian ridge regression is used to fuse the training results of single models under different subdata sets. In the simulation experiment, the AUC value of the NN-ATT-Bayesian-Stacking model constructed in this paper reaches 0.9675, and the distribution of the prediction results is ideal. Thus, this model can make an accurate and reliable evaluation in the financing and loan scenarios of SMEs, which is of great significance to the financing, risk management, and financial service supervision of SMES. In addition, this model has a broad application perspective and possesses practical significance and theoretical research value.

## Figures and Tables

**Table 1:** Correlation coefficient selection.

Accuracy	Metadata (%)	Subset 1 (%)	Subset 2 (%)
Random forest	91.139	89.255	91.051

**Table 2:** Tree model selection.

Accuracy	Metadata (%)	Subset 3 (%)	Subset 4 (%)
Random forest	91.139	91.139	88.911

**Table 3:** Experimental environmental configuration.

OS	Ubuntu 16.04.5 LTS 64 bits
CPU	E5-2620 v3 @ 2.40 GHz,x2
GPU	GTX TITAN *X* (pascal) GPU 12 GB,x2
CUDA	9.0.176
TensorFlow	2.0.0
Scikit-learn	0.23.1

**Table 4:** AUC value of base model.

Classifier	Raw	Subset 1	Subset 2	Subset 3	Subset 4
Logistic	0.8980	0.8992	0.8925	0.8956	0.8962
Linear	0.9034	0.8880	0.8855	0.8868	0.8869
Bayesian	0.8921	0.8942	0.8892	0.8908	0.8986
LGB	0.9266	0.9248	0.9155	0.9255	0.9231
XGB	0.9285	0.9304	0.9238	0.9294	0.9185
NN	0.9198	0.9109	0.9123	0.9114	0.9044
NN-ATT	0.9035	0.9101	0.9003	0.9017	0.9019
CNN	0.9257	0.9214	0.9158	0.9101	0.9137

**Table 5:** AUC value of stacking model.

Classifier	Raw	Subset 1	Subset 2	Subset 3	Subset 4
NN-XGB	0.9299	0.9176	0.9287	0.9191	0.9203
NN-ATT-XGB	0.9299	0.9217	0.9318	0.9128	0.9194
CNN-XGB	0.9299	0.9275	0.9267	0.9188	0.9232
NN-LGB	0.9266	0.9195	0.9167	0.9128	0.9225
NN-ATT-LGB	0.9266	0.9127	0.9173	0.9208	0.9229
CNN-LGB	0.9266	0.9215	0.9161	0.9292	0.9228

**Table 6:** AUC value of fusion model.

Classifier	Raw	Subset
Bayesian-stacking	0.9475	0.9645
Linear–stacking	0.9644	0.9644
Logistic–stacking	0.9673	0.9673
CNN-Bayesian-stacking	0.9489	0.9675
CNN-Linear–stacking	0.9489	0.9674
CNN-Logistic–stacking	**0.9477**	**0.9727**
NN-Bayesian-stacking	0.9489	0.9709
NN-Linear–stacking	0.9489	0.9658
NN-Logistic–stacking	0.9477	0.9775
NN-ATT-Bayesian-stacking	0.9489	0.9675
NN-ATT-Linear–stacking	0.9489	0.9675
NN-ATT-Logistic–stacking	0.9492	0.9711

## Data Availability

The data used to support the findings of the study are available from the corresponding author upon request. The author's e-mail address is zhangleicqjtu@163.com.
